# Epidemics and food systems: what gets framed, gets done

**DOI:** 10.1007/s12571-020-01072-5

**Published:** 2020-07-08

**Authors:** Stuart Gillespie

**Affiliations:** International Food Policy Research Institute, Washington DC, USA

**Keywords:** COVID-19, Epidemic, Food system, Nutrition, Resilience, Transformation

## Abstract

This brief article aims to interrogate some widely used concepts in framing the interactions between disease epidemics, food systems and nutrition, with a particular focus on the COVID-19 crisis. How should we conceptualize vulnerability in such situations – both with regard to viral exposure and to subsequent nutrition-relevant impacts of epidemics and responses (including lockdowns)? Is it possible to simultaneously pursue strategies aimed at strengthening resilience and driving transformation (‘building back better’)? What type of framing and conceptualization can help illuminate entry points and options for responding effectively to interacting crises? In addressing these questions, it’s important to re-visit lessons from past attempts to address the impacts of epidemics on food and nutrition security.

The way we respond to a crisis is governed by the way we frame and describe the threat or shock. The COVID-19 pandemic has generated a whole new language (e.g. lockdown, physical distancing) and a dusting-down of terms and concepts from past crises. Such concepts are important as they help us construct narratives that explain how problems emerge, how they may be addressed and how future crises may be prevented.

Whether linked to health, economic, climate or conflict shocks, resilience has come to be seen as a useful organizing principle -- a mobilizing metaphor that transcends disciplines and sectors. The less common notion of resistance (to the initial risk of viral exposure) may also be helpful in developing preventive responses. Third, recent discourse on food system transformation (Willett et al. 2019; McDermott and de Brauw 2020; Herrero et al. 2020) is being used to frame approaches to ‘build back better’.

Do such concepts help improve understanding, communication and the development of comprehensive responses to the interactions between COVID-19, food systems and nutrition?

Two decades ago, I co-founded the RENEWAL program at IFPRI to investigate the implications of the AIDS epidemic for food and nutrition security in eastern and southern Africa. The first step was to convene local stakeholders to discuss how to conceptualize and approach this challenge. Building on the evidence available at that time, a conceptual map was developed, highlighting the drivers of HIV infection, and the impacts of AIDS epidemics, from macro (environmental) to micro (individual) levels. The map was then used to identify and locate potential responses (prevention, care, treatment, mitigation) as they applied to the drivers and impacts (Loevinsohn and Gillespie 2003).

Drawing on this approach, we can begin to develop a simplified version for COVID-19, food and nutrition, as shown in Fig. 1. The non-exhaustive list of factors in the four columns derives from emerging evidence of association (on which it’s not possible to elaborate in this short article).

**Fig. 1:**
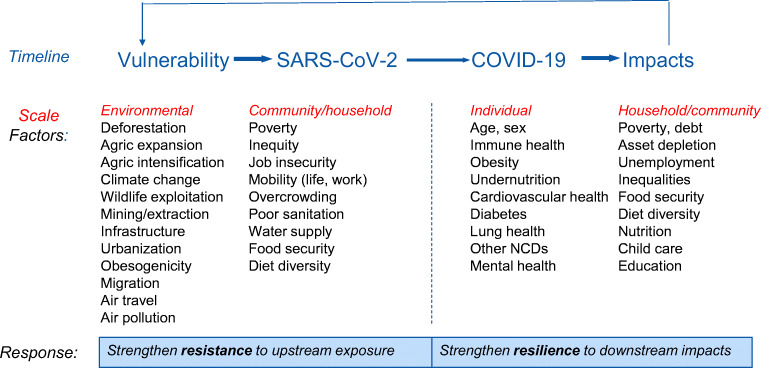
COVID-19: vulnerability, resistance and resilience

Reading Fig. 1 from left to right, we can see how vulnerability to certain drivers (operating from environmental to individual levels) conditioned the spread of the SARS-COV-2 virus and determined who was exposed to, and infected by it. Appropriate responses here will be preventive, aimed at strengthening *resistance* to exposure to the virus (by controlling or reducing the drivers listed in the first two columns). The front-line of resistance to any viral infection is an individual’s immune system (shown at the centre). After infection, we are in the realm of *resilience*, with key factors listed in the two right-hand columns. Important here are a set of individual-level characteristics (including obesity and undernutrition) that we now know will determine the severity of COVID-19 disease and ultimately the likelihood of dying from it. Beyond such individual factors lie a series of impacts at different scales (household, community etc) that will kick in over different time spans. Many such impacts are indirect in that they result from the immediate responses to the epidemic (especially lockdowns). Depending on the metric used (e.g. lives saved), it’s quite likely that such indirect impacts will be significantly greater than the impact of the viral infection itself (Robertson et al. 2020). Finally, Fig. 1 shows the feedback loop from impacts to future vulnerabilities. Poverty and inequity, for example, can drive upstream vulnerability to viral exposure, as well as be exacerbated by the downstream effects of the epidemic – potentially setting up a vicious cycle.

Alongside resilience, we see another emerging discourse that argues for the need to jettison business-as-usual in a post-COVID world, to create a ‘new normal’ and to ‘build back better’. This sounds very like transformation -- quite different to coping.

This leads us to an important question. When applied to food systems in the midst of a pandemic, can these two goals – resilience and transformation – co-exist?

To address this, we need to dig deeper. There are many definitions of resilience (Pelletier et al. 2016; Ansah et al. 2019). Most refer to the ability – in the face of a shock or stress -- to recover or bounce back to a past state. A type of buoyancy, elasticity or toughness in the face of adversity -- the capacity to weather the storm, to cope. The Intergovernmental Panel on Climate Change, for example, defines resilience as the ‘ability of a system and its component parts to anticipate, absorb, accommodate, or recover from the effects of a hazardous event in a timely and efficient manner’ (IPCC 2012). In the face of COVID-19, at an individual level, resilience is ultimately the ability to survive. For households, it’s the ability to withstand multiple social and economic impacts.

Some definitions have been criticized as ignoring issues of power, agency and social justice. In the context of AIDS epidemics, many similarly viewed the notion of ‘coping’ as a cop-out – an escape from the challenge of confronting how people’s capabilities were being stunted (Barnett and Whiteside 2002). Resilience may not even be an appropriate goal if it reinforces an inequitable *status quo*. It is quite conceivable, moreover, for a household to demonstrate resilience but at a very high cost -- for example, if the maintenance of household food security derives from the hazardous/arduous work of women who become ill or undernourished themselves and/or unable to care for young children whose growth falters.

We therefore need to ask questions about equity, about the cost of resilience, and who pays. We need to consider scale (individual, household, community) and timeline (e.g. does resilience endure?). We are rapidly learning how the COVID-19 pandemic is not only exposing different forms of inequity but also amplifying them. Individuals who pay the highest price for national resilience are often front-line health and social care workers who -- day after day, separated from their own families for weeks on end -- put their lives at risk to keep people alive. The AIDS epidemic also spread along societal fault lines, widening them in the process (Barnett and Whiteside 2002).

An important conceptual step forward was taken by Bene et al. (2014) when they incorporated power and agency into a broader definition of resilience. Beyond the capacity to cope, they highlight the capacity to adapt, and even to transform as being encapsulated within a spectrum of resilience. The ability to absorb a shock (cope) ensures stability, which in turn provides the potential for incremental adjustments (adaptation) and even transformational change in this more inclusive framing. In practical terms, in the face of COVID-19, for example, a regular cash transfer to poor households could ensure stability, raise risk horizons and potentially open up more livelihood options and space for innovation (Gilligan 2020).

This could apply to households and communities -- and it could apply it to health and food systems. Systems-thinking helps us break out of sectoral silos, but we need to retain a wide-angled lens to see the connections *between* systems. We can, for example, see how certain food systems in which wild animals, domestic animals and humans are in close proximity in wet markets may be vulnerable to zoonotic emergence (Bett et al. 2020). The SARS-COV-2 virus first crossed species and now it has cut across entire systems. Emerging from a food system, it has gone on to overwhelm health systems, and to undermine global economic systems in a way that’s not been seen for more than a century.

Just as systems interact, so can epidemics. ‘Syndemics’ are synergistic epidemics that overlap in time and space, interact with each other, and share common underlying drivers. The 2019 Lancet Commission report on the global syndemic of obesity, undernutrition and climate change described these interactions in depth (Swinburn et al. 2019). The COVID-19 pandemic is a new addition to the mix – a short-wave shock that overlaps and interacts with these three long-wave crises. Take obesity for example – we know that the obesity epidemic is being driven, in part, by the accessibility and affordability of ultra-processed foods and beverages -- the ‘obesogenic environment’ (Rauber et al. 2020; Tan et al. 2020). Evidence is also mounting on how obesity confers a greater risk of poor outcomes of COVID-19 disease, including death (Docherty et al. 2020). Compounding this, lockdowns are contributing to a rise in obesity due to reduced physical activity and difficulties in sourcing a healthy diet – especially among the poorest households (Pietrobelli et al. 2020).

In sum, resilience can be a useful common goal across sectors and systems -- so long as it is treated comprehensively, and so long as it includes an analysis of agency and equity. It is possible to strive for resilience and to pave the way for transformation into a more sustainable, more equitable future. These two goals are not mutually exclusive. But it will require actions that seek to progressively strengthen all three resilience capacities (absorptive, adaptive and transformative) at multiple levels (individual, household, community).
